# Sprouting Enhances Submergence Tolerance in Rice by Promoting Glutathione Biosynthesis and Turnover

**DOI:** 10.3390/antiox14121387

**Published:** 2025-11-21

**Authors:** Mei Wang, Na Kuang, Ziyi Mao, Shangfeng Zhou, Zhixuan Liu, Ke Chen, Licheng Liu, Jingbo Xu, Lifeng Wang, Haoyu Lu

**Affiliations:** 1 State Key Laboratory of Hybrid Rice, Hunan Hybrid Rice Research Center, Hunan Academy of Agricultural Sciences, Changsha 410125, China; 15574808897@163.com (M.W.); kuangna19@163.com (N.K.); 18673654756@163.com (Z.M.); zhixuanliu@hunaas.cn (Z.L.); chenke57@163.com (K.C.); liulicheng713@163.com (L.L.); jb68@263.net (J.X.); 2 Hunan Institute of Plant Protection, Changsha 410125, China; sfzhou7389@hunaas.cn; 3 Yuelushan Laboratory, Changsha 410128, China

**Keywords:** rice, submergence stress, soaking and sprouting, glutathione, antioxidant system

## Abstract

Submergence stress is a major constraint in direct-seeded rice production. This study investigated the effect and biochemical mechanism of sprouting, a traditional agronomic practice, on improving submergence tolerance in rice. Our findings demonstrate that sprouting is an effective seed treatment that significantly enhances the plant’s ability to withstand flooding. Specifically, 48 h sprouting increased shoot height and root length by 163% and 423%, respectively, in the YLYJ48 variety under 6-day submergence. Sprouting upregulated the activity of glutathione reductase (GR) and the expression of its related genes, thereby significantly promoting the biosynthesis of glutathione (GSH). GSH content in seeds increased from 64.86 µg g^−1^ FW (0 h) to 83.00 µg g^−1^ FW (48 h) in HZ, and from 82.14 to 92.52 µg g^−1^ FW in YLYJ48. This process provides critical antioxidant protection for seedlings to implement a rapid “escape strategy,” ultimately enhancing their submergence tolerance. Functional verification showed that seed soaking with exogenous GSH (0.1%) effectively improved submergence tolerance by increasing antioxidant reserves. Exogenous GSH treatment elevated shoot height by approximately 50% in both HZ and YLYJ48 varieties under submergence. Field trials further demonstrated that exogenous GSH application significantly enhanced seedling establishment rates by 30–35% and improved seedling growth traits under submergence tolerance stress across multiple rice varieties. This study reveals part of the mechanism by which sprouting enhances submergence tolerance by influencing glutathione metabolism, offering practical strategies for flood-resilient direct-seeded rice cultivation.

## 1. Introduction

Rice (*Oryza sativa* L.) is a vital global food crop, and its stable production is crucial for world food security [1]. During the early sowing and seedling stages, flooding caused by heavy rainfall, poor land leveling, or inadequate drainage can completely submerge rice seeds, severely inhibiting germination and seedling establishment and ultimately leading to substantial yield losses [2]. Furthermore, intentional flooding is widely adopted in practice as an environmentally friendly weed control measure, which undoubtedly poses additional challenges to the submergence tolerance of rice seedlings [3,4]. Therefore, elucidating the physiological mechanisms of rice response to submergence stress and developing effective mitigation strategies are of great importance for ensuring stable rice yields.

Studies have found that rice employs two distinct survival strategies in response to submergence stress: one is the “quiescence strategy,” which conserves energy consumption to wait out the stress, and the other is the “escape strategy,” characterized by rapid organ elongation to flee the flooded environment. However, regardless of the strategy adopted, the plant’s antioxidant capacity has been demonstrated to be critical for its submergence tolerance [5,6,7]. Submergence stress triggers a burst of reactive oxygen species (ROS) accumulation in plant tissues; excessive ROS attack polyunsaturated fatty acids in cell membranes, inducing lipid peroxidation and consequent oxidative damage [8,9]. To maintain cellular redox homeostasis, plants have evolved a sophisticated antioxidant defense system. This system includes enzymatic components such as superoxide dismutase (SOD), catalase (CAT), ascorbate peroxidase (APX), glutathione reductase (GR), and glutathione peroxidase (GPX), as well as non-enzymatic antioxidants like ascorbate (ASA) and glutathione (GSH) [10,11].

Among these antioxidants, GSH, a central endogenous antioxidant, plays a critical role in regulating plant redox balance and stress responses [12]. Its function is particularly crucial for scavenging excess ROS under the hypoxic conditions induced by submergence [13]. GSH is synthesized from glutamate, cysteine, and glycine in two sequential steps catalyzed by γ-glutamylcysteine synthetase (GSH1) and glutathione synthetase (GSS). In the antioxidant cycle, GSH directly scavenges ROS and is oxidized to oxidized glutathione (GSSG). The latter can be regenerated back to GSH by GR in an NADPH-dependent manner, thereby maintaining the dynamic balance of the GSH/GSSG ratio. Recent studies have demonstrated that enhancing GSH levels, either through exogenous application or endogenous biosynthesis, can effectively improve rice tolerance to various environmental stresses [14,15,16].

To improve germination and seedling establishment in rice, “cuiya” (sprouting) has been widely adopted by Chinese farmers as a traditional agronomic practice since its systematic documentation in the 6th-century manual *Qimin Yaoshu*. While some studies have indicated that seed soaking and sprouting can significantly enhance the antioxidant capacity of seeds in plants such as rice, soybean, and quinoa, research on the ability of sprouting to improve submergence tolerance in rice remains scarce [17,18,19]. Whether and how sprouting enhances submergence tolerance by augmenting antioxidant capacity requires in-depth investigation.

This study aims to systematically elucidate the biochemical and molecular mechanisms by which traditional sprouting technology enhances submergence tolerance in rice, with a specific focus on its regulatory effect on the glutathione metabolism pathway. By integrating biochemical phenotyping and transcriptomic data, we reveal for the first time that sprouting treatment remodels glutathione metabolism, thereby strengthening the seedling’s overall antioxidant defense capacity. Furthermore, exogenous GSH application validated the central role of glutathione in mitigating submergence stress, providing a theoretical basis and technical support for the modern application of this traditional agronomic practice.

## 2. Materials and Methods

### 2.1. Plant Growth Conditions and Treatments

The submergence treatment was applied according to the method described by Lu et al. (2023) with minor modifications [20]. The detailed procedure was as follows: Seeds were surface-sterilized with 1.5% (*v*/*v*) sodium hypochlorite for 20 min, rinsed thoroughly with double-distilled water (ddH_2_O), and then soaked in pure water at 26 °C for 24 h. For the exogenous GSH soaking experiments, GSH was added to the pure water at this step to create concentration gradients of 0 (pure water control), 0.001%, 0.01%, 0.1%, and 1% (*w*/*v*) GSH, with soaking lasting 24 h. After soaking, seeds of uniform size were selected and transferred into cell PCR plates (Hunan Simock Biotechnology Co., Ltd., Changsha, China) with bottom openings (aperture radius 1.2 mm). The seeded PCR plates were then placed in germination boxes lined with moist filter paper. Sprouting treatment was conducted at 26 °C in a constant temperature water bath in the dark for different durations (0, 12, 24, 36, and 48 h).

Following the sprouting treatment, the PCR plates were transferred to a submergence device. For the normal hydroponics group, the water level was maintained at the height of the seed embryo. For the submergence tolerance group, the water level was maintained 9 cm above the seeds. Submergence devices were placed in an artificial climate chamber (FH-1200, HiPoint, Kaohsiung, Biobase, Jinan, China) set at 26 °C, 50% humidity, a 14/10 h light/dark cycle, and a light intensity of 6000 lx. Pure water was replenished every two days to maintain the respective water levels. The specific experimental design is summarized in Table 1.

The following rice varieties were used in this study: Huazhan (HZ), Yunliangyoujiu 48 (YLYJ48), Huanghuazhan (HHZ), Zhongzao 39 (ZZ39). All rice seeds used in this study were provided by Hunan Golden Nongfeng Seed Industry Co., Ltd. (Changsha, China). Reduced glutathione (GSH) was purchased from Shandong Keyuan Pharmaceutical Co., Ltd. (Jinan, China).

### 2.2. Measurement of Rice Phenotypic and Biochemical Parameters

Seedling phenotypic data were measured using a vernier caliper. Samples were collected separately from seeds and their corresponding seedlings (after 6 days of submergence stress) subjected to 0, 24, and 48 h sprouting treatments and 0.1% GSH treatment. The samples were quickly placed onto absorbent paper to remove surface water, promptly transferred to liquid nitrogen for rapid freezing, and stored at −80 °C for subsequent analysis.

The activities of SOD, peroxidase (POD), and CAT were determined using the nitroblue tetrazolium (NBT) photochemical reduction method, the guaiacol method, and the hydrogen peroxide (H_2_O_2_) ammonium molybdate catalytic decomposition method, respectively. The content of malondialdehyde (MDA) was measured using the thiobarbituric acid (TBA) colorimetric method [21]. GR and GPX activities were assayed by the NADPH oxidation method and the coupled NADPH oxidation method, respectively. The contents of GSH and GSSG were quantified using the DTNB colorimetric method and the enzymatic recycling method with 2-vinylpyridine derivatization, respectively [22]. All biochemical parameters were determined by Wuhan ProNets Testing Technology Co., Ltd., Wuhan, China.

### 2.3. RNA Extraction, Library Construction, RNA Sequencing, and Data Analysis

Shanghai OE Biotech Co., Ltd. (Shanghai, China) used the Illumina sequencing platform to sequence and analyze transcriptomes. The RNA-seq analysis included three independent biological replicates per treatment, with each replicate comprising a pooled sample of multiple seedlings. Raw reads were quality-controlled using fastp (v0.20.1; https://github.com/OpenGene/fastp, accessed on 26 August 2025) and FastQC (v0.11.9; Babraham Bioinformatics, Cambridge, UK) to remove low-quality bases. High-quality reads were then aligned to the *Oryza sativa* L. genome using HISAT2 (v2.1.0; Johns Hopkins University, Baltimore, MD, USA). Gene expression levels were quantified with HTSeq-count (v0.11.2; European Molecular Biology Laboratory, Heidelberg, Germany) and normalized as FPKM (fragments per kilobase of transcript per million mapped reads). Differential expression analysis was conducted using DESeq2 (version 1.22.2; Bioconductor, http://bioconductor.org/, accessed on 26 August 2025), and genes were considered significantly differentially expressed if they met the following thresholds: an average FPKM ≥ 10 across replicates (to ensure detection reliability), an absolute log_2_ fold change > 1, and an adjusted *p*-value (FDR) < 0.05 corrected by the Benjamini–Hochberg procedure. Functional enrichment analysis of the identified genes was performed based on the Kyoto Encyclopedia of Genes and Genomes (KEGG) and Gene Ontology (GO) databases [5].

### 2.4. Field Trial Verification

The experiment employed a randomized complete block design. Four rice varieties (YLYJ48, HZ, HHZ, and ZZ39) were subjected to two different seed soaking treatments: soaking in pure water (0 h) and soaking in 0.1% (*w*/*v*) GSH solution (0.1% GSH). Each treatment was replicated three times, with individual plots measuring 0.04 m^2^ (20 cm × 20 cm). Sowing was conducted on 1 May 2025, in Yanwuzhou Village, Liuyang County, Changsha City, Hunan Province (LON 113.23 E; LAT 23.06 N), on a typical local red soil with medium fertility and acidic pH. The field had been plowed and leveled prior to sowing to ensure maximum surface uniformity. Additionally, according to local cultivation practices, compound fertilizer (Sinochem Fertilizer Co., Ltd., Beijing, China) was applied as a base fertilizer at a rate of 50 kg ha^−1^. One-hundred treated seeds were directly sown in each plot, followed by artificial flooding to a 5 cm water depth, which was maintained for 16 days with daily checks and replenishment at 6:00 a.m. and 6:00 p.m. to impose submergence stress. Rice shoot height and root length data were recorded after the 6-day stress period. The number of established seedlings was investigated 15 days after sowing. A seedling was considered successfully established if it had more than two true leaves, an erect stem, and no deformities. The seedling establishment rate was calculated as follows: Establishment Rate (%) = (Number of Established Seedlings/100) × 100%. Meteorological data are provided in Supplementary Table S4.

### 2.5. Statistical Analysis

In this study, all data are presented as the mean ± standard deviation (SD). Statistical analyses were performed using SPSS version 20.0 (IBM SPSS Statistics, Chicago, IL, USA). One-way analysis of variance (ANOVA) followed by Tukey’s post hoc test was used for multiple comparisons to control the family-wise error rate. Principal component analysis (PCA) was employed to visualize the overall structure and grouping patterns in the transcriptomic dataset. For comparisons between two groups, a two-tailed *t* test was applied. Data organization was performed using Microsoft Excel 2019 (Microsoft, Redmond, WA, USA). Figures were generated with GraphPad Prism 8 (GraphPad Software Inc., San Diego, CA, USA) and the OE Biotech online platform (https://cloud.oebiotech.com/(accessed on 26 August 2025)).

## 3. Results

### 3.1. Phenotypic and Biochemical Changes of Sprouted Seedlings Under Submergence Stress

**Figure 1:**
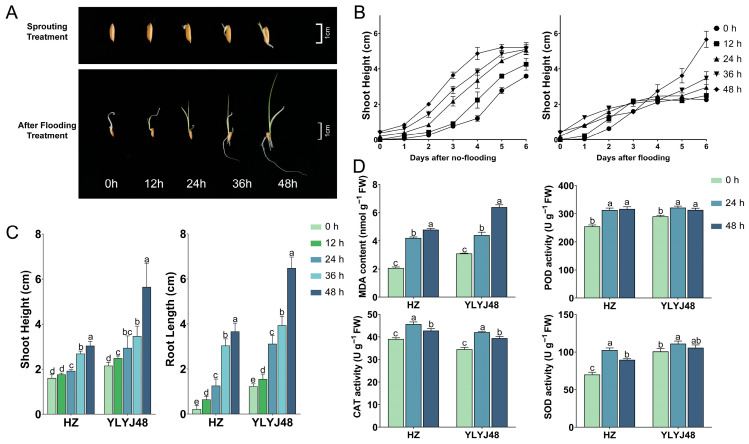
Phenotypic and biochemical responses of rice seedlings to submergence stress under different sprouting treatments. (**A**) Phenotype of YLYJ48 seedlings after 6-day submergence following 0–48 h sprouting treatments. (**B**) Shoot height dynamics of YLYJ48 under normal and submerged conditions during 6-day treatment. (**C**) Shoot height and root length of HZ and YLYJ48 varieties after 6-day submergence. (**D**) MDA content and CAT, SOD, and POD activities in HZ and YLYJ48 seedlings after 6-day submergence. HZ, Huazhan; YLYJ48, Yunliangyoujiu 48 (rice cultivars). The time points (0 h, 12 h, 24 h, 36 h, and 48 h) indicate the duration of sprouting treatment initiated after a uniform 24 h water soak. MDA, malondialdehyde; CAT, catalase; SOD, superoxide dismutase; POD, peroxidase. The error bars in panels (**B**–**D**) are the SD of triplicate experiments (*n* = 3). Different letters indicate significant differences (one-way ANOVA, Tukey’s test, *p* < 0.05).

To evaluate the effect of sprouting on rice growth under submergence stress, we compared the shoot height dynamics of the YLYJ48 variety under different sprouting durations (0, 12, 24, 36, and 48 h) in conventional hydroponic conditions and submergence tolerance (Figure 1A,B). Under conventional hydroponic conditions, the shoot growth dynamics across different sprouting treatments were similar, indicating that sprouting had a limited effect on the final shoot height or elongation rate in the absence of stress. However, under submergence stress, the growth dynamics of the treatment groups showed clear differences. The shoot elongation in the 0 h and 12 h treatments was largely arrested after 3 days of submergence, whereas that in the 24 h and longer sprouting treatments continued. Among these, the 48 h treatment exhibited a noticeably higher shoot elongation rate compared to the other groups. Combined with phenotypic data from the conventional rice variety HZ under submergence stress (Figure 1C), we found that seedling phenotypes in both genetically distinct rice varieties (YLYJ48 and HZ) were significantly promoted with increasing sprouting duration. In YLYJ48, the 48 h treatment increased shoot height and root length by 163% and 423%, respectively, compared to the 0 h treatment. A similar promoting effect was observed in the HZ variety.

However, this rapid “escape” growth was accompanied by severe oxidative damage. Biochemical analysis revealed that the MDA content in seedlings of both varieties increased significantly with prolonged sprouting time under submergence stress (Figure 1D). Assays of three key antioxidant enzymes—POD, CAT, and SOD—showed that the activities of these enzymes in the 24 h treated seedlings of both varieties were significantly superior to those in the 0 h treatment. Although the enzyme activities in the 48 h treatment were somewhat reduced compared to the 24 h treatment, they remained higher than in the 0 h treatment. These results indicate that sprouting treatment of rice seeds can alter subsequent growth dynamics and biochemical responses under complete submergence. Detailed information is provided in Supplementary Table S5.

### 3.2. Transcriptome Analysis Reveals That Sprouting Regulates the Glutathione Metabolic Pathway

#### 3.2.1. RNA Sequencing (RNA-Seq) Analysis and Identification of Differentially Expressed Genes (DEGs)

**Figure 2:**
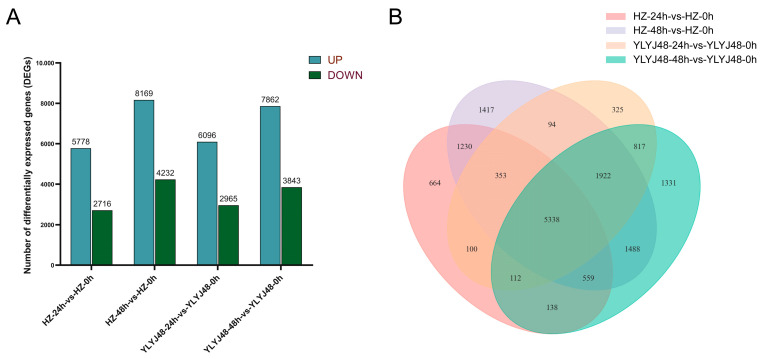
Differentially expressed genes (DEGs) in seeds under different sprouting treatments. (**A**) Number of DEGs in two rice varieties (YLYJ48 and HZ) at 24 h and 48 h of sprouting compared to the 0 h control. (**B**) Unique and shared DEGs among different sprouting treatments. Sample comparisons are denoted as [Variety]-[Sprouting Duration]-vs.-[Variety]-0h, where HZ: Huazhan; YLYJ48: Yunliangyoujiu 48; 0 h, 24 h, and 48 h: duration of sprouting treatment after a 24 h water soak (e.g., HZ-24h-vs.-HZ-0h represents Huazhan at 24 h sprouting compared to its 0 h control). UP, upregulated DEGs; DOWN, downregulated DEGs.

RNA-seq was performed on 18 rice seed samples from YLYJ48 and HZ varieties subjected to three sprouting treatments (0, 24, and 48 h). Sequencing generated 768 million raw reads, from which 746 million clean reads were obtained with Q30 scores above 95.27% and an average GC content of 51.48%. All samples showed genome mapping rates exceeding 92%, confirming data quality suitable for reliable analysis (Table S1). Correlation analysis between samples confirmed high reproducibility within treatments (R^2^ > 0.90). Principal component analysis (PCA) revealed clear separation among sprouting treatments, with biological replicates clustering tightly within each group (Figure S1). Differential expression analysis identified substantial transcriptional changes across treatments. Compared to HZ-0h, HZ-24h contained 8494 DEGs (5778 upregulated, 2716 downregulated) while HZ-48h showed 12,401 DEGs (8169 upregulated, 4232 downregulated). Similarly, YLYJ48-24h and YLYJ48-48h exhibited 9061 (6096 upregulated, 2965 downregulated) and 11,705 DEGs (7862 upregulated, 3843 downregulated), respectively, relative to YLYJ48-0h (Figure 2), The number of differentially expressed genes (DEGs) showed variation depending on both treatment duration and genetic background.

#### 3.2.2. GO and KEGG Pathway Enrichment Analysis of DEGs from Different Sprouting Treatments

**Figure 3:**
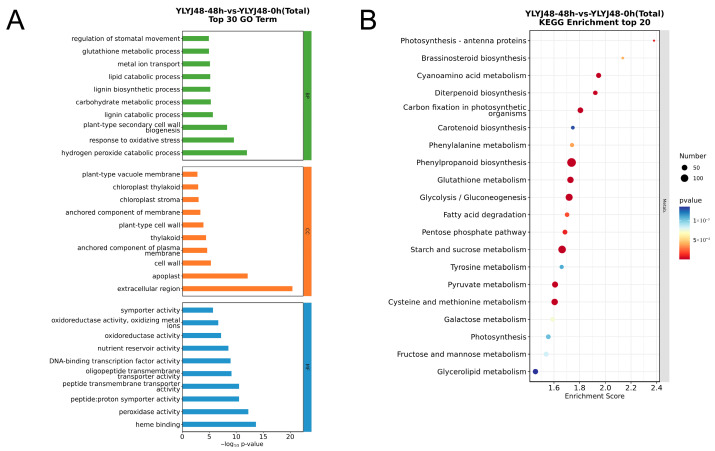
Functional enrichment analysis of differentially expressed genes between YLYJ48 seeds subjected to 48 h and 0 h sprouting treatments. (**A**) GO enrichment analysis showing the top 30 most significant terms. (**B**) KEGG pathway enrichment analysis showing the top 20 most significant pathways.

Through integrated analysis of DEGs from comparisons between HZ-24h-vs.-HZ-0h, HZ-48h-vs.-HZ-0h, YLYJ48-24h-vs.-YLYJ48-0h, and YLYJ48-48h-vs.-YLYJ48-0h, we found that the two rice varieties exhibited certain similarities in gene expression patterns after sprouting treatment. To systematically analyze the biological functions of these differentially expressed genes, we conducted GO and KEGG enrichment analyses (Figure 3 and Figure S2).

According to the results from GO term enrichment, the differentially expressed genes were categorized into three functional classes: biological process, cellular component, and molecular function. Under 24 h sprouting treatment, both varieties were significantly enriched in antioxidant-related processes such as peroxidase activity (GO:0004601), response to oxidative stress (GO:0006979), hydrogen peroxide catabolic process (GO:0042744), response to reactive oxygen species (GO:0000302), and glutathione metabolic process (GO:0006749). In the 48 h treatment, in addition to enrichment in the aforementioned antioxidant processes like hydrogen peroxide catabolic process (GO:0042744), response to oxidative stress (GO:0006979), and glutathione metabolic process (GO:0006749), it also induced processes such as plant-type secondary cell wall biogenesis (GO:0009834), lipid catabolic process (GO:0016042), and carbohydrate metabolic process (GO:0005975). These changes indicate that under 48 h sprouting treatment, rice seeds begin extensive formation of secondary cell walls to ensure mechanical strength, water transport, and stress resistance. The coordinated changes in lipid and carbohydrate metabolism reflect the energy demands of cell growth.

KEGG pathway analysis revealed that under 24 h sprouting treatment, the differentially expressed genes in both varieties were significantly enriched in glutathione metabolism (osa00480) and its synthesis-related pathway—cysteine and methionine metabolism (osa00270). Pathways related to secondary metabolite synthesis, including phenylpropanoid biosynthesis (osa00940), diterpenoid biosynthesis (osa00904), and carotenoid biosynthesis (osa00906), were also enriched with a large number of differentially expressed genes. These pathways are involved in plant functions such as disease resistance, insect resistance, UV protection, and photoprotection. Carbohydrate metabolism pathways serving as energy supply, including starch and sucrose metabolism (osa00500), glycolysis/gluconeogenesis (osa00010), and galactose metabolism (osa00052), were also highly enriched. Under 48 h sprouting treatment, the glutathione metabolism network remained highly active. These transcript-level changes provide a molecular explanation for the enhanced antioxidant capacity induced by sprouting treatment.

#### 3.2.3. Sprouting Regulates Key Genes in the Glutathione Metabolism Pathway of Rice

**Figure 4:**
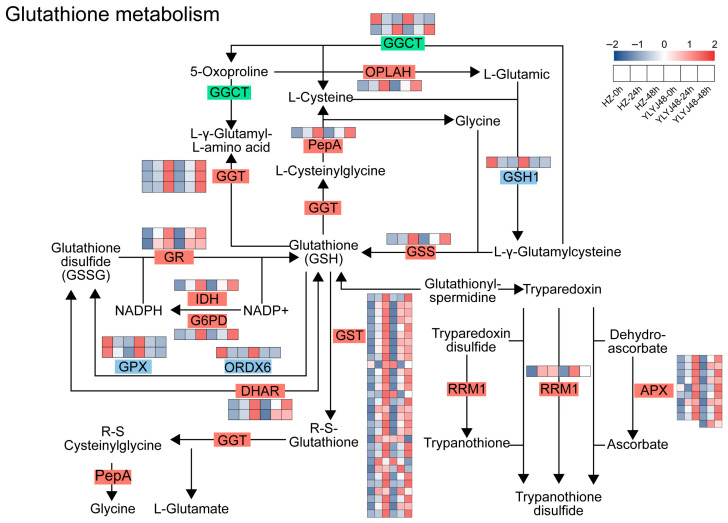
Glutathione metabolism pathway in rice seeds after sprouting treatment. Heatmap shows the expression of DEGs under different sprouting durations. Gene expression is presented as Z-score. The abbreviated enzymes are defined as follows: GGCT, γ-glutamyl cyclotransferase; OPLAH, 5-oxoprolinase; PepA, probable aminopeptidase P; GGT, γ-glutamyltransferase; GSS, glutathione synthetase; GR, glutathione reductase; IDH, isocitrate dehydrogenase; G6PD, glucose-6-phosphate 1-dehydrogenase; GPX, glutathione peroxidase; ORDX6, oxidoreductase 6; DHAR, dehydroascorbate reductase; GST, glutathione S-transferase; RRM1, ribonucleoside-diphosphate reductase large subunit; APX, ascorbate peroxidase. The glutathione metabolism pathway is presented based on the KEGG reference map (map00480), following the style of Zhang et al. [23]. The heatmap displaying gene expression Z-scores is original data from this study.

To investigate the regulatory mechanism of sprouting treatment on the antioxidant system in rice, we performed pathway enrichment analysis of differentially expressed genes (DEGs). A total of 60 DEGs were enriched in the glutathione metabolism pathway (Figure 4 and Table S2). Compared with the 0 h control, the expression of multiple key genes in this pathway changed significantly with prolonged sprouting time.

Genes involved in glutathione synthesis (*GSS*), reduction and regeneration (*GR*, *IDH*, and *G6PD*), transport and conjugation (*GST*), and the ascorbate–glutathione cycle (*DHAR* and *APX*) were significantly upregulated with extended sprouting treatment. However, against this overall upregulation trend, the expression of *GPX* and *ORDX6* was significantly downregulated. These two genes encode enzymes that utilize GSH as a substrate to scavenge peroxides and play critical roles in the glutathione redox cycle. Their expression patterns, opposite to that of *GR*, may substantially influence the content of endogenous antioxidant GSH and the GSH/GSSG balance in plants.

### 3.3. Effects of Sprouting Treatment on Basal Biochemical Indicators in Rice Seeds

**Figure 5:**
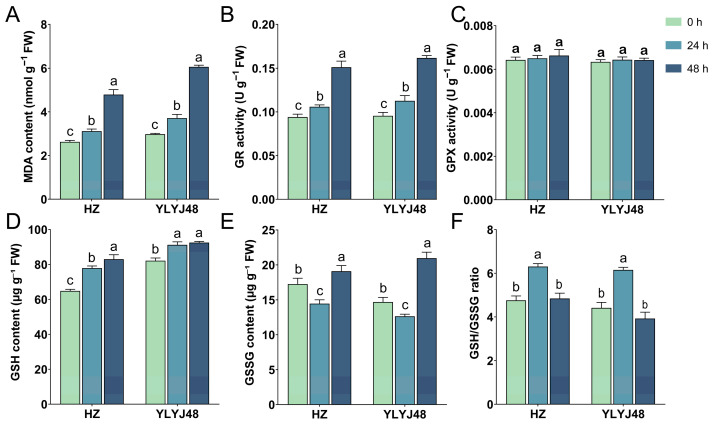
Biochemical parameters of rice seeds under different sprouting durations. (**A**) Malondialdehyde (MDA) content. (**B**) Glutathione reductase (GR) activity. (**C**) Glutathione peroxidase (GPX) activity. (**D**) Reduced glutathione (GSH) content. (**E**) Oxidized glutathione (GSSG) content. (**F**) GSH/GSSG ratio. The error bars in panels (**A**–**F**) are the SD of triplicate experiments (*n* = 3). Different letters indicate significant differences (one-way ANOVA, Tukey’s test, *p* < 0.05).

Given the significant impact of sprouting on the glutathione metabolism pathway, we measured MDA content, GR and GPX activities, and GSH and GSSG levels in seeds subjected to different sprouting durations (0 h, 24 h, and 48 h). The results showed that sprouting significantly altered the intrinsic biochemical status of the seeds (Figure 5). Under non-stress conditions, MDA content gradually increased with prolonged sprouting time, indicating that extended metabolic activity during sprouting itself imposed a certain degree of oxidative stress on the seeds. Meanwhile, sprouting significantly enhanced the basal activity of GR. In contrast, GPX activity remained relatively stable across treatments, with no significant differences observed. Endogenous GSH content showed an increasing trend with longer sprouting time. However, GSSG content did not change uniformly: in both varieties, the basal GSSG level was lowest after 24 h of sprouting and highest after 48 h. The GSH/GSSG ratio showed no significant difference between the 0 h and 48 h treatments, while the 24 h treatment was significantly higher than both. These findings demonstrate that sprouting treatment enhances endogenous GSH levels and regeneration capacity in seeds, thereby providing critical antioxidant support for seedlings to sustain rapid growth under submergence stress. Detailed information is provided in Supplementary Table S5.

### 3.4. Functional Verification and Field Application of Exogenous Glutathione in Enhancing Submergence Tolerance

**Figure 6:**
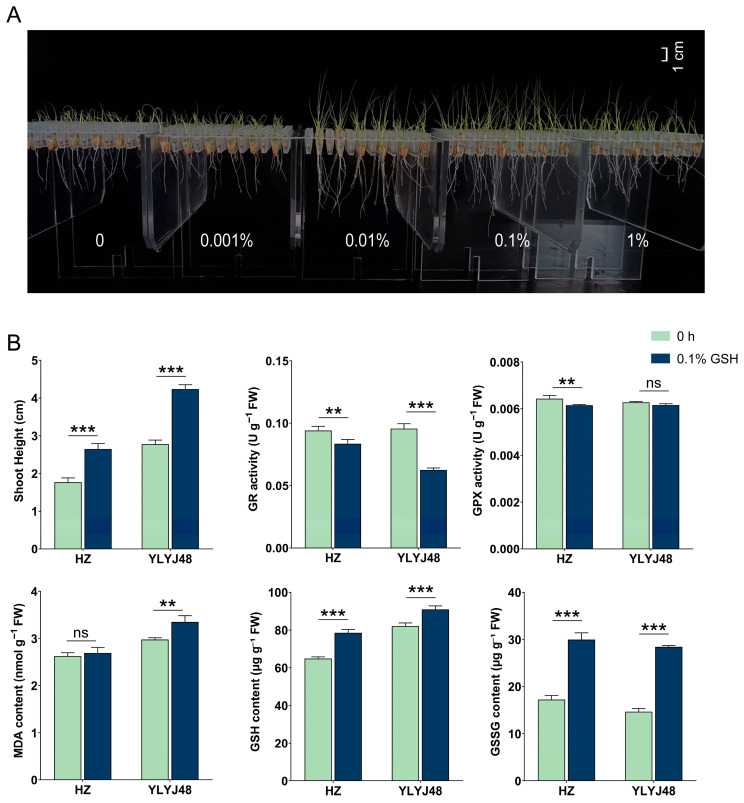
Effects of exogenous glutathione on submergence tolerance in rice. (**A**) Phenotype of YLYJ48 seedlings after 6-day submergence following treatment with different GSH concentrations (0, 0.001%, 0.01%, 0.1%, and 1% (*w*/*v*)). (**B**) Basal biochemical parameters of seeds after 0.1% (*w*/*v*) GSH treatment and shoot height of the corresponding seedlings after 6-day submergence in two rice varieties (HZ and YLYJ48). The error bars in panels (**B**) are the SD of triplicate experiments (*n* = 3). ns: no significant difference; ** *p* < 0.01, and *** *p* < 0.01 (two-tailed *t* tests).

To verify glutathione’s role in enhancing submergence tolerance, we optimized the GSH concentration using YLYJ48 rice (Figure 6). Phenotypic screening identified 0.1% as optimal, while a higher concentration (1%) showed inhibition. Subsequent experiments with two varieties revealed that 0.1% GSH treatment significantly elevated both GSH and GSSG content while reducing GR and GPX activities. This treatment enhanced shoot elongation by approximately 50% after submergence in both varieties.

Field trials across four genetically diverse rice varieties demonstrated broad applicability. The 0.1% GSH treatment consistently improved submergence tolerance, significantly promoting shoot and root growth across all tested varieties. Most notably, it dramatically increased seedling establishment rates under field conditions, with improvements ranging from approximately 30% to over 35% absolute increase depending on variety. Detailed information is provided in Supplementary Table S5.

## 4. Discussion

This study demonstrates for the first time that sprouting—a simple, low-cost seed treatment—enhances submergence tolerance in rice by regulating glutathione metabolism and improving antioxidant capacity.

### 4.1. Sprouting Enhances Submergence Escape Through Improved Antioxidant Capacity

In this experiment, rice seedlings from the sprouting treatment elongated under complete submergence. Their growth rate increased with longer sprouting duration. The well-developed aerenchyma of rice supports partial submergence tolerance; however, complete submergence severely compromises survival. Faster emergence through the water surface represents a manifestation of submergence tolerance [24]. However, this “escape strategy” centered on rapid elongation entails significant biochemical costs. In our study, the 48 h sprouting treatment group with the most vigorous growth suffered severe oxidative damage. The accelerated metabolism and growth processes generate substantial ROS, while moderate ROS levels act as signaling molecules activating genes that promote internode elongation, driving accelerated underwater shoot extension [25,26]. Thus, the 48 h group exhibited rapid elongation phenotypically while biochemically sustaining more severe membrane damage due to ROS accumulation.

For successful escape submergence, rice must counteract oxidative damage from both flooding stress and rapid growth. Through regulation of the endogenous antioxidant system, damage caused by excessive ROS accumulation can be mitigated, thereby promoting plant growth. In our study, seedlings from the 24 h sprouting treatment demonstrated significantly higher activities of key antioxidant enzymes (POD, SOD, and CAT) compared to controls, facilitating better growth under submergence [7]. Integrating biochemical data from sprouted seeds, we propose that sprouting treatment establishes elevated baseline GSH content and GR activity even before stress exposure, indicating robust antioxidant reserve and regeneration capacity. This enhancement of overall antioxidant potential provides fundamental support for growth during submergence stress.

### 4.2. Regulation of Glutathione Metabolism by Sprouting Contributes to Submergence Tolerance in Rice

Our integrated transcriptomic and biochemical analyses reveal that sprouting enhances submergence tolerance by inducing a pre-adaptive reconfiguration of glutathione metabolism. This metabolic reprogramming does not represent a simple upregulation of all components but is characterized by synergistic enhancement: on one hand, the upregulation of biosynthesis and regeneration genes (e.g., *GSS*, *GR*), coupled with the transcriptional downregulation of oxidation genes (e.g., *GPX*, *ORDX6*), collectively promotes the net accumulation of GSH; on the other hand, the coordinated induction of utilization genes (e.g., *APX*, *GSTs*) enhances the seed’s functional capacity for ROS scavenging and xenobiotic metabolism.

Consistent with previous reports that plants modulate antioxidant expression to mitigate waterlogging stress [27,28], this study observed that the expression and enzymatic activity of the *GR* gene (*LOC_Os03g06740*) significantly increased with sprouting duration, facilitating the reduction of GSSG to GSH under submergence stress and thereby improving overall antioxidant capacity [29,30]. Notably, GPX, a key enzyme in the glutathione system, functions in peroxide detoxification and redox signaling [31,32,33,34]; although its gene expression was downregulated during sprouting, its enzymatic activity remained stable at the biochemical level. This phenomenon is consistent with previous reports [35]. We propose that this observed phenomenon reflects a resource optimization strategy, whereby plants reallocate metabolic resources between growth and defense depending on environmental conditions [36]. During non-stress periods, maintaining—rather than further elevating—GPX activity helps to prioritize limited energy toward fundamental seed germination metabolism and the synthesis of antioxidants such as GSH. This approach simultaneously enables the preservation of baseline ROS levels, which can function as signaling molecules to promote germination. Consequently, plants accumulate sufficient reducing power to maintain redox homeostasis when subsequently confronted with stress conditions.

The upregulation of both APX and GST genes during sprouting suggests a coordinated enhancement of the rice seedling’s defense arsenal. APX, a hub enzyme in the ascorbate–glutathione cycle, enables specific detoxification of H_2_O_2_, while GSTs confer dual functions in antioxidant defense and xenobiotic detoxification [37,38]. Building on evidence that transgenic overexpression of APX or GST genes enhances environmental stress tolerance in rice, our findings indicate that sprouting treatment acts as a natural priming strategy [39,40]. This pre-sowing intervention proactively induces a similar molecular configuration, thereby equipping the seedlings with a more robust antioxidant and detoxification capacity to confront impending submergence stress.

### 4.3. Practical Applications of Sprouting Treatment and Exogenous Glutathione

Traditional soaking and sprouting technology can effectively enhance the submergence tolerance of rice, which is of great significance for promoting direct seeding technology in flood-prone areas. Both seed sprouting and seed priming are pre-sowing seed treatment technologies that can improve germination performance and antioxidant capacity. However, the complexity and high cost of seed priming technology limit its widespread application in staple crops like rice. In contrast, sprouting technology offers the advantages of simple operation and extremely low cost, making it an irreplaceable option for resource-poor impoverished areas and small-scale farmers. Nevertheless, in large-scale production, sprouted seeds with exposed coleoptiles are susceptible to damage during mechanical sowing, limiting their application in large-scale direct seeding of rice [41].

Addressing the core contradiction of sprouting technology—“clear effectiveness but inconvenient application”—this study identified the key endogenous substance responsible for enhancing submergence tolerance: GSH. Based on this, we proposed and validated an innovative solution: simulating the biochemical effects of sprouting through exogenous GSH treatment, thereby avoiding the associated costs and sowing risks. Experiments showed that exogenous GSH treatment significantly increased the content of GSH and GSSG in seeds, effectively ensuring seedling growth under submergence conditions—a result validated in field trials.

More importantly, exogenous GSH treatment demonstrates broad application prospects. Previous studies have shown that exogenous GSH can serve as a seed priming agent to effectively repair aged seeds and improve their germination rate under non-stressed conditions [42,43]. This study found that an extremely low concentration (0.01%) of GSH treatment significantly enhanced submergence tolerance in rice, indicating substantial cost advantages of this strategy. Furthermore, we observed that sufficient exogenous GSH may suppress the activity of endogenous GR through a feedback mechanism [44,45], which might explain why excessively high GSH concentrations (e.g., 1%) resulted in reduced effectiveness. It is noteworthy that even at such high concentrations, the submergence tolerance phenotype remained significantly superior to the untreated control group, demonstrating that GSH possesses a wide safety margin in practical applications.

In summary, this study provides two alternative strategies to address submergence stress in direct-seeded rice: in impoverished areas or small-scale farms, low-cost traditional sprouting technology can be directly adopted; in large-scale agricultural production, exogenous GSH treatment is recommended to enhance submergence tolerance at lower cost and with greater flexibility.

### 4.4. Implications for Breeding and Integrated Crop Management

These findings offer valuable insights for both breeding programs and agronomic practices. For breeding, the identified key genes in the glutathione metabolism pathway (e.g., *GSS*, *GR*) could serve as potential markers for selecting varieties with enhanced intrinsic submergence tolerance [46]. Furthermore, our successful use of exogenous GSH suggests a promising complementary approach: GSH priming could be integrated with the cultivation of existing submergence-tolerant cultivars [47]. Such a combination strategy is expected to provide more robust and reliable protection for direct-seeded rice production in flood-prone environments, leveraging both genetic improvement and agronomic intervention.

## 5. Conclusions

This study confirms that sprouting regulates the expression of glutathione metabolism-related genes, enhances the content of glutathione, and improves overall antioxidant defense capability, significantly promoting rice seedling growth. Furthermore, exogenous application experiments verify the practical effectiveness of GSH in mitigating submergence stress in agricultural production; these results support the integration of sprouting in small-scale farming and GSH seed treatment in large-scale, direct-seeded rice production.

## Figures and Tables

**Table 1:** Experimental design.

Experiment	Soaking Treatment	Sprouting Duration	Subsequent Stress Treatment
Effect of Sprouting on Submergence Tolerance	Pure water	0, 12, 24, 36, 48 h	Normal hydroponics and submergence (9 cm, 6 d)
GSH Concentration Screening	0 (Pure water), 0.001%, 0.01%, 0.1%, 1% GSH	0 h	Submergence (9 cm, 6 d)
GSH Functional Verification	0 (Pure water), 0.1% GSH	0 h	Submergence (9 cm, 6 d)
Field Trial Verification	0 (Pure water), 0.1% GSH	0 h	Artificially created 5 cm water layer (6 d)

Note. All experiments were performed with three independent biological replicates. For phenotypic assessment, data were collected from multiple individual seedlings per replicate and are presented as mean values. For biochemical assays and transcriptomic analysis, each biological replicate consisted of a pooled sample derived from multiple seedlings subjected to the same treatment. GSH, glutathione.

## Data Availability

The original contributions presented in this study are included in the article/Supplementary Material. Further inquiries can be directed to the corresponding author.

## Supplementary Materials

The following supporting information can be downloaded at https://www.mdpi.com/article/10.3390/antiox14121387/s1, Figure S1: Quality assessment of RNA-seq data from all samples; Figure S2: Functional enrichment analysis of differentially expressed genes from additional sprouting treatment comparisons; Figure S3: Effects of exogenous 0.1% GSH treatment on seedling establishment under field conditions; Table S1: Transcriptome sequencing sample quality results; Table S2: Expression levels (FPKM) and Z-scores of differentially expressed genes in the glutathione metabolism pathway; Table S3: Phenotypic responses of rice seedlings to exogenous GSH treatments under submergence stress; Table S4: Meteorological conditions during the field trial period; Table S5: Raw data for Figure 1, Figure 5 and Figure 6.
