# Supplementary figures and images for: Sprouting Enhances Submergence Tolerance in Rice by Promoting Glutathione Biosynthesis and Turnover
Source: Antioxidants (Basel). 2025 Nov 21;14(12):1387. doi: 10.3390/antiox14121387 (PMC12729939; doi:10.3390/antiox14121387)

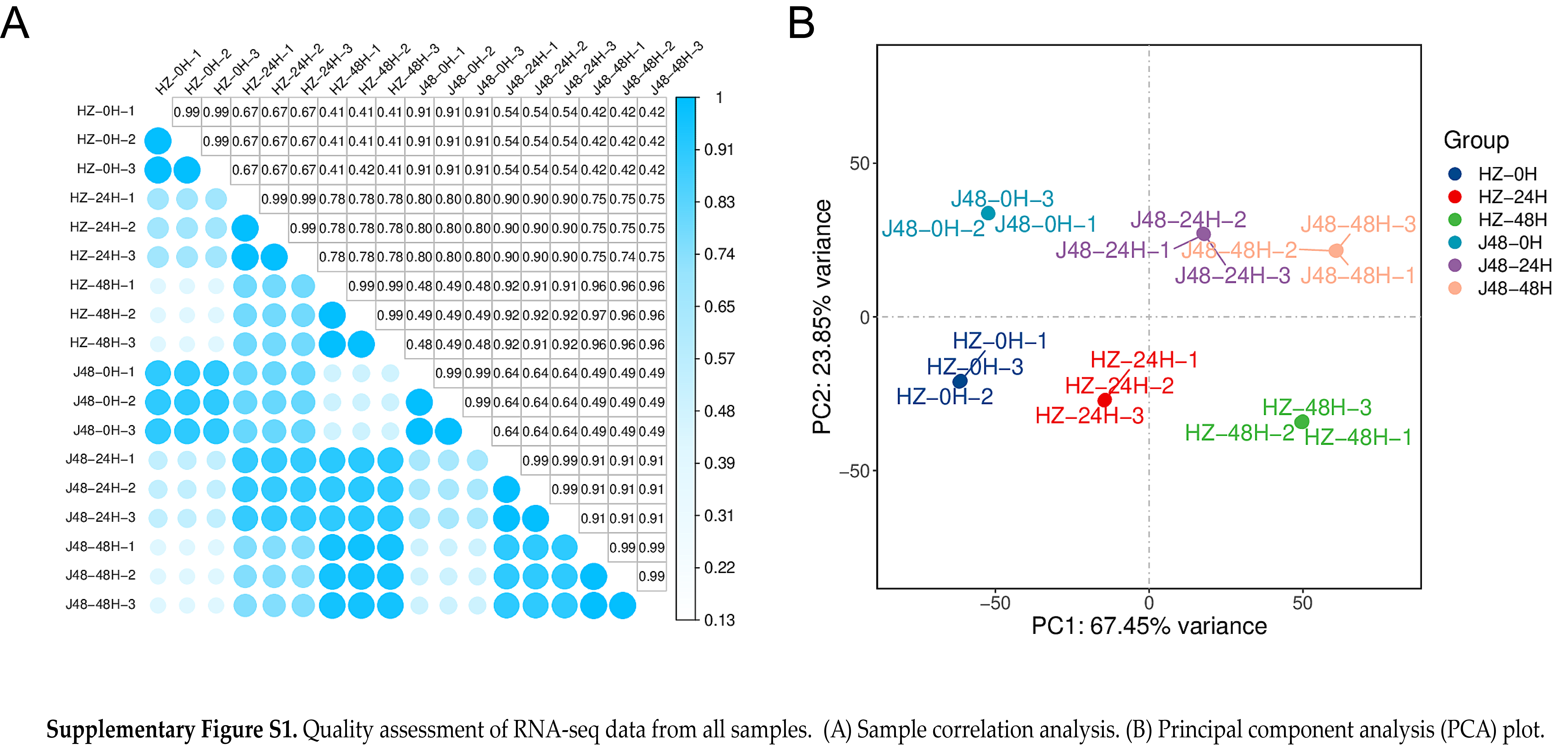

Supplement: Supplementary file 1 [file antioxidants-14-01387-s001.zip › Supplementary Figure S1.png]

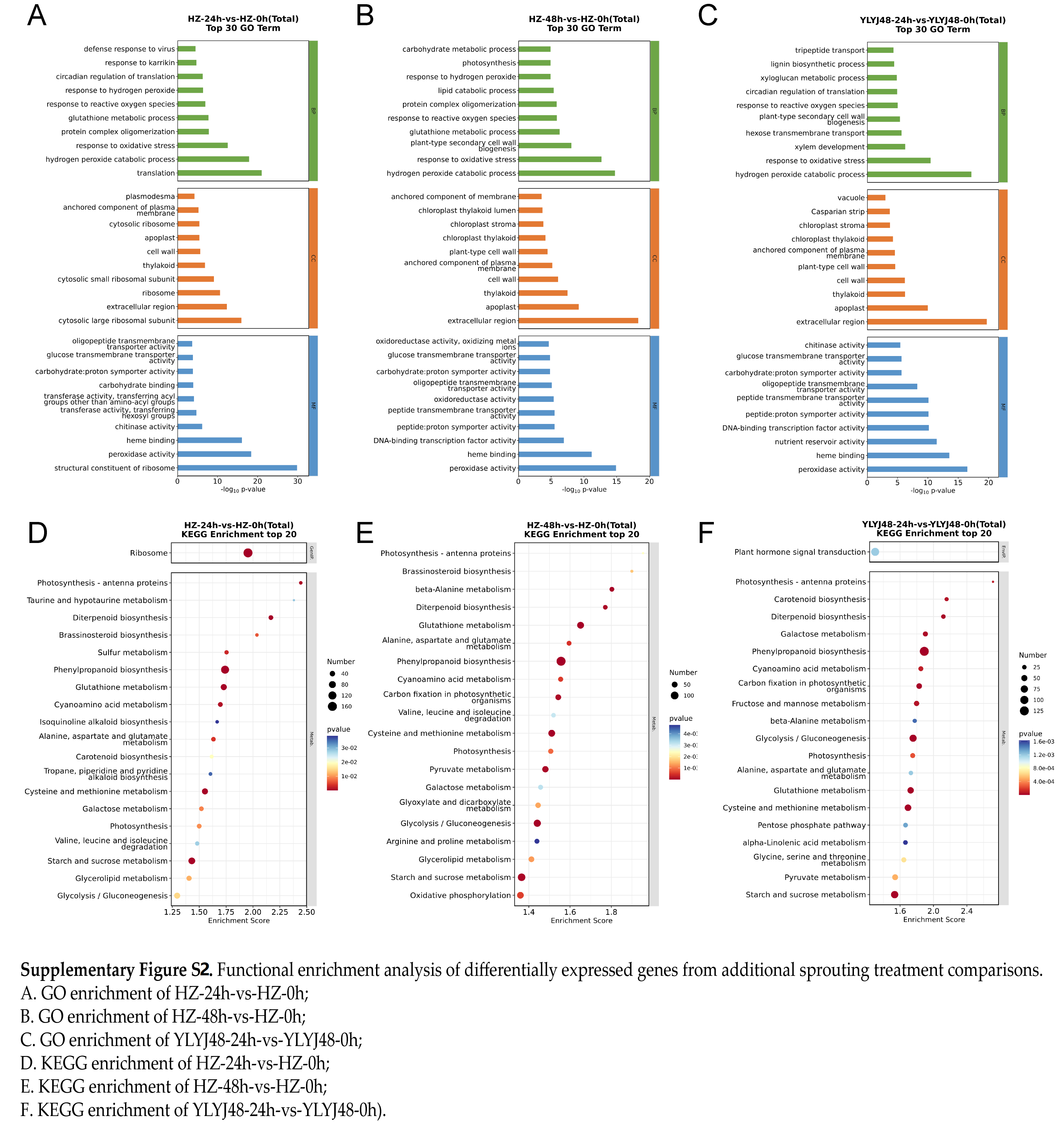

Supplement: Supplementary file 1 [file antioxidants-14-01387-s001.zip › Supplementary Figure S2.png]

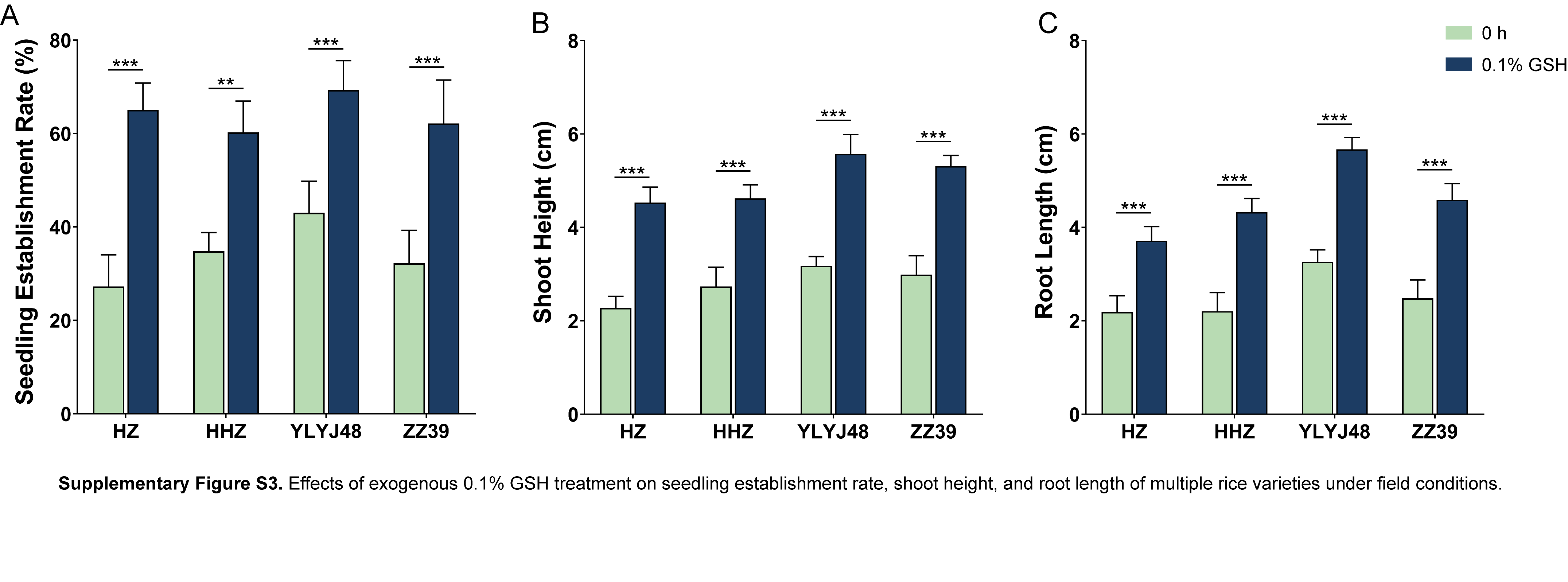

Supplement: Supplementary file 1 [file antioxidants-14-01387-s001.zip › Supplementary Figure S3.png]
